# Insights from Chilean NCDs Hospitalization Data during COVID-19

**DOI:** 10.3390/medicina60050770

**Published:** 2024-05-07

**Authors:** Jaime Andrés Vásquez-Gómez, Chiara Saracini

**Affiliations:** 1 Centro de Investigación de Estudios Avanzados del Maule (CIEAM), Vicerrectoría de Investigación y Postgrado, Universidad Católica del Maule, Talca 3460000, Chile; jvasquez@ucm.cl; 2 Centro de Investigación en Neuropsicologia y Neurociencias Cognitivas (CINPSI Neurocog), Facultad de Ciencias de la Salud, Universidad Católica del Maule, Talca 3460000, Chile

**Keywords:** COVID-19, hospitalizations, chronic diseases, physical activity, NCDs

## Abstract

The COVID-19 pandemic has affected the lifestyles of people of all ages, conditions and occupations. Social distance, remote working, changes in diet and a lack of physical activity have directly and indirectly affected many aspects of mental and physical health, particularly in patients with many comorbidities and non-communicable diseases (NCDs). In our paper, we analyzed COVID-19 hospitalized and non-hospitalized cases according to comorbidities to assess the average monthly percentage change (AMPC) and monthly percentage change (MPC) using open access data from the Chilean Ministry of Science, Technology, Knowledge and Innovation. As expected, the infection mainly affected patients with comorbidities, including cardiovascular risk factors. The hospitalized cases with obesity and chronic lung disease increased throughout the period of June 2020–August 2021 (AMPC = ↑20.8 and ↑19.4%, respectively, *p* < 0.05), as did all the non-hospitalized cases with comorbidities throughout the period (AMPC = ↑15.6 to ↑30.3 [*p* < 0.05]). The increases in hospitalizations and non-hospitalizations with comorbidities may be associated with physical inactivity. A healthy lifestyle with regular physical activity may have had a protective effect on the COVID-19 severity and related events in the post-pandemic period, especially for the NCD population.

## 1. Introduction

The global pandemic of COVID-19 that started in December 2019 has radically changed what we once thought of as “normal” life. Since the Spanish flu of 1918 —the first pandemic to be adequately documented [1]—there have been other pandemics in history, but none of them have had the same impact on modern life as COVID-19 [2]. This includes more recent pandemics like SARS in 2002, H1N1 swine flu in 2009, and MERS in 2012. The sixth pandemic on record affected almost every nation on earth, forcing leaders to enact measures that had a significant global impact on the socio-cultural, political and economic spheres [3].

The virus caused a significant loss of human life and political and economic assets, with over 200 million people infected and around 5 million deaths. The global trade was projected to decline by 5.3% in 2020, and the global economic growth was expected to have decreased by −3.4% to −7.6% annually [3]. A number of countries imposed severe restrictions on the freedom and movement of their populations between 2020 and 2021. These measures included national lockdowns, quarantines and bans on social gatherings, even among family members.

There is no doubt that this pandemic brought about extreme changes in people’s lifestyles and human relationships, which in turn affected the concept of the workplace, social gatherings, communication, arts and entertainment [4]. At the same time, COVID-19 seemed to mainly affect people with a previously compromised clinical picture, hitting hardest and with more aggressive symptoms regarding patients suffering from cardiometabolic diseases (hypertension, diabetes, dyslipidaemia, obesity, cardiopulmonary disorders, etc.) and certainly the immunocompromised [5]. It is likely that infections have increased with the chronic deterioration of the population’s health in the pre-pandemic phase as some retrospective studies have reported an increase in the number of comorbidities [6], an increase in body mass index (BMI) associated with diabetes [7] and the likelihood of chronic diseases compared to a decade ago [8]. These clinical conditions and comorbidities are considered to be associated with higher levels of economic well-being and a more ‘relaxed’ lifestyle and are referred to as “non-communicable diseases” (NCDs).

In Latin America, Chile is one of the countries with the highest economic status [9,10], and, as the per capita income has increased, so have the health problems related to NCDs [11]. Studies report that, in Chile between 1990 and 2019, before the pandemic, high BMI, high blood glucose levels and hypertension were prevalent in the population [12], that some of the cardiovascular risk factors were often associated with other risk factors [13] and that the development of type 2 diabetes was particularly associated with physical inactivity [14]. The problem of a lack of physical activity (PA) in Chile is such that, according to the latest National Health Survey of 2016–17, the percentage of sedentary lifestyle in the Chilean population ranged from 88.6 to 86.7% from 2009–10 to 2016–17, in which the last year sedentary lifestyle was 83% for men and 90% for women, and, in addition, 27.1% of the population was physically inactive. There were also increases in body fat, suspected diabetes and heart attacks [15]. Thus, the triad of COVID-19–comorbidities–physical inactivity has had an impact that has been insufficiently studied in Chile but which could be an example to be projected over time from a theoretical approach to promote studies evaluating the impact of PA as a protective factor, on the one hand, and to promote interventions to improve the general state of health of the population through the promotion of healthy lifestyles in all the countries suffering from these trends towards an increase in pathologies of this type on the other.

We believe that it is very important to consolidate the evidence on the relationship between chronic diseases and the effects of COVID-19 infection because it could have a transcendental impact on public health not only in Chile but in any country, providing information that could be used in future actions in the post-pandemic phase by the relevant health institutions, and, at the same time, prove useful to private and public health centers regarding the virtuous interaction they had during the pandemic and, finally, have an impact on the direct beneficiaries, that is, the general population, taking into account the obstacles and opportunities offered by the pandemic scenario. For these reasons, the objective of this analysis was to evaluate the evolution of the COVID-19 inpatient and outpatient cases according to the presence of comorbidities in the Chilean population and, as already suggested in the literature, to promote vaccinations and lifestyle changes mediated by the practice of PA as a protective and complementary treatment for post-pandemic infections, not only in Chile but also globally.

## 2. Materials and Methods

We analyzed data published by the Ministry of Science, Technology, Knowledge and Innovation of the Republic of Chile on patients hospitalized by COVID-19 in the private and public health systems, as well as non-hospitalized cases during the pandemic period, whose anonymized records were publicly available in the GitHub online repository [16], accessible until at least September 2021 [17]. Ethical approval from an academic ethics committee and/or health service medical board was not required, as well as written informed consent. However, the study adhered to the tenets of the Declaration of Helsinki (2013).

The datasets analyzed corresponded to anonymized cases (published without information on sex, age or chronological age range, basic demographic data or anthropometric characteristics) in the pandemic period between June 2020 (first month recorded in the GitHub online repository) and August 2021, where each of the cases could have one or more of the following comorbidities: hypertension, diabetes, obesity, asthma, cardiovascular disease, chronic lung disease, chronic heart disease, immunocompromised and chronic liver disease.

The trend of COVID-19 hospitalized and non-hospitalized cases by comorbidity was evaluated using Joinpoint Regression v. 4.9.0 (USA), calculating the average monthly percentage change (AMPC) and the monthly percentage change (MPC) for the entire pandemic period and for each shorter period, respectively, according to the oscillations of the cases detected by the statistical program. In this way, statistically significant or nonsignificant fluctuations during the period were described, characterized by the intersection points between intermediate periods on the trend curve and by percentages accompanied by their respective 95% confidence intervals (CIs), considering statistical significance at a *p*-value of less than 5%. Finally, Student’s *t*-test for independent samples was used to evaluate significant differences (*p* < 0.05) between hospitalized and non-hospitalized cases.

To assess sensitivity and quantify potential biases, we first calculated the assumption of heteroscedasticity for the association between the two conditions (response: hospitalized; exploratory: not hospitalized) with the Breusch–Pagan (BP) test and then performed an odds ratio (OR) risk analysis to assess the likelihood of people being hospitalized in the COVID-19 pandemic periods (exposed group: June–August 2020; unexposed: September 2020 to August 2021).

## 3. Results

**Figure 1:**
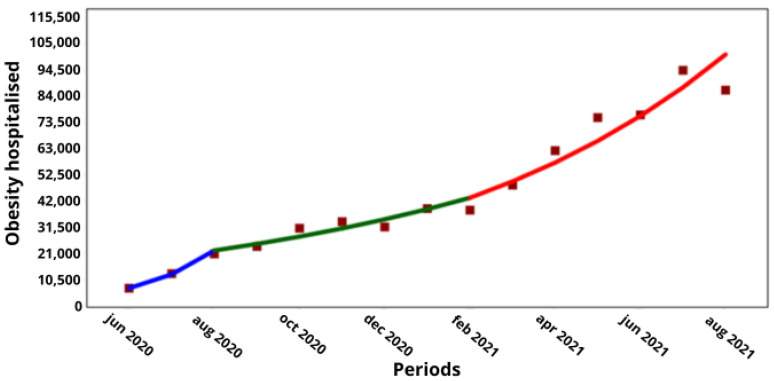
Trend in hospitalized cases of people with obesity by COVID-19 in Chile. (**X axis**) = periods and junctions. (**Y axis**) = number of cumulative cases. Different colors of the trend line indicate the three periods (blue = Period 1; green = Period 2; Red = Period 3).

**Figure 2:**
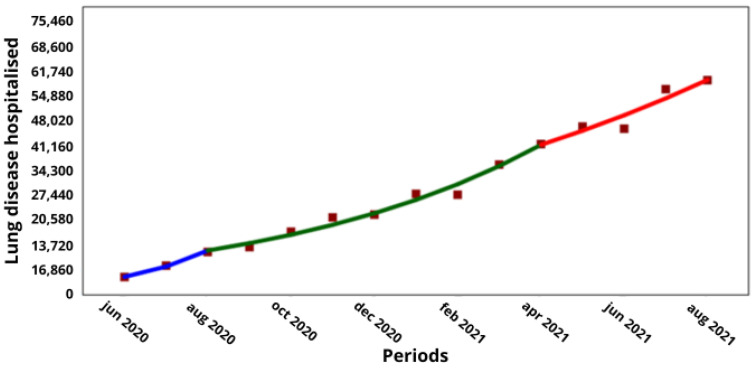
Trend in hospitalized cases of people with chronic lung disease by COVID-19 in Chile. (**X axis**) = periods and junctions. (**Y axis**) = number of cumulative cases. Different colors of the trend line indicate the three periods (blue = Period 1; green = Period 2; Red = Period 3).

The COVID-19 hospitalized and non-hospitalized cases during the pandemic period are shown in Table 1 and Table 2, respectively. All the hospitalized cases increased significantly in the overall period between June 2020 and August 2021, and also in the first period between June and August 2020 for all the associated comorbidities. There were significant increases during a marked gap (period 2) between August 2020 and May 2021 and in a third period from November 2020 to August 2021, but only for some of the comorbidities. The comorbidities that showed statistically significant increases throughout the pandemic period, in the three intermediate periods, i.e., with two join points, were obesity and chronic lung disease with an AMPC of ↑20.8 (*p* < 0. 05) and ↑19.4% (*p* < 0.05), respectively (Table 1), and, for obesity, an MPC of ↑73.3 (*p* = 0.002), ↑11.2 (*p* = 0.005) and ↑16.2 (*p* < 0.001) in the first, second and third interim periods, respectively (Figure 1). In chronic lung disease, increases of ↑56.9 (*p* = 0.004), ↑16.5 (*p* < 0.001) and ↑9.4% (*p* = 0.035) were observed in the three consecutive interim periods (Figure 2).

There was a clear and significant trend in the increase in non-hospitalized cases with comorbidities throughout the pandemic period (June 2020 and August 2021) and in the first interim period (June–August 2020) for all the comorbidities. There was also an increase in the non-hospitalized cases with comorbidities in the third period between December 2020 and August 2020 (Table 2), but it is noteworthy that there was no significant increase in the second period of August–December 2020 for any comorbidity (Table 2).

**Figure 3:**
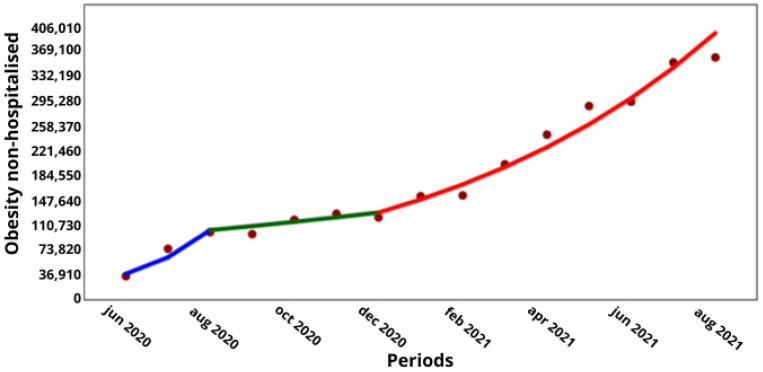
Trend in non-hospitalized cases of people with obesity by COVID-19 in Chile. (**X axis**) = periods and junctions. (**Y axis**) = number of cumulative cases. Different colors of the trend line indicate the three periods (blue = Period 1; green = Period 2; Red = Period 3).

**Figure 4:**
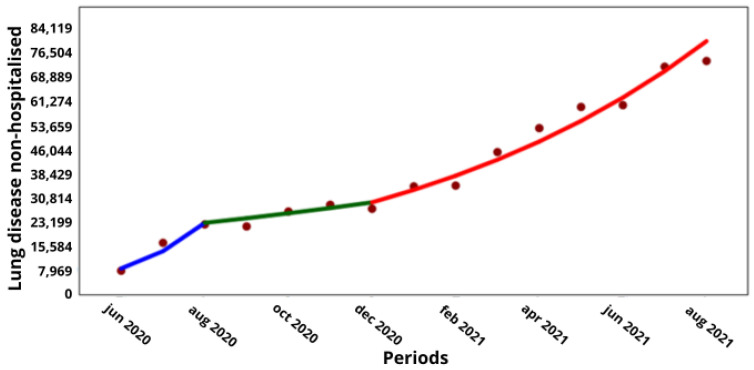
Trend in non-hospitalized cases of people with chronic lung disease by COVID-19 in Chile. (**X axis**) = periods and junctions. (**Y axis**) = number of cumulative cases. Different colors of the trend line indicate the three periods (blue = Period 1; green = Period 2; Red = Period 3).

With regard to the comorbidities of obesity and chronic lung disease, which were the only comorbidities with a significant increase in COVID-19 hospitalizations during the pandemic and in the intervening period, a comparison with the corresponding non-hospitalized cases showed that obesity increased from 38,835 to 97,669 hospitalized cases over 6 months (↑16%; *p* < 0.001), while the non-hospitalized cases increased from 122,834 to 362,022 cases over 8 months (↑15%; *p* < 0.001; Figure 1 and Figure 3, respectively), while the non-hospitalized cases increased from 122,834 to 362,022 cases over 8 months (↑15%; *p* < 0.001; Figure 1 and Figure 3). Meanwhile, the hospitalized cases with lung disease increased from 11,938 to 42,032 over 8 months (↑16.5%; *p* < 0.001), and the non-hospitalized cases increased from 27,642 to 74,535 over 8 months (↑13.3%; *p* < 0.001; Figure 2 and Figure 4, respectively).

Finally, we calculated *t*-tests of both hospitalized and non-hospitalized cases to evaluate the magnitude of trends for each comorbidity. As can be seen in Table 3, there were differences in cases between the hospitalized and non-hospitalized subjects, except for chronic kidney disease. In the entire period analyzed (June 2020 to August 2021), the highest number of cases corresponded to the non-hospitalized subjects with comorbidities, a phenomenon that could hypothetically be attributed to the positive effects of COVID-19 vaccination.

The association between the hospitalized and non-hospitalized subjects with asthma proved to be homoscedastic (BP = 2.62; *p* = 0.11), as well as hospitalized and non-hospitalized individuals with CHD (BP = 2.99; *p* = 0.083), with diabetes (BP = 0. 29; *p* = 0.58), with CVD (BP = 3.34; *p* = 0.067), with CLiD (BP = 3.31; *p* = 0.068), with HBP (BP = 3.03; *p* = 0.081), with ICP (BP = 1.23; *p* = 0. 26), with CND (BP = 1.35; *p* = 0.24), with obesity (BP = 1.35; *p* = 0.24), with CLuD (BP = 2.51; *p* = 0.11) and with CKD (BP = 3.22; *p* = 0.072). Therefore, there was no relationship between the explanatory variables and the errors.

The people studied in June–August 2020, during the full stage of COVID-19 pandemic, were 9% more likely to be hospitalized for asthma than in the subsequent pandemic periods between September 2020 and August 2021 (OR = 1.09; CI: 1.07–1.11). There were also higher odds for CHD (OR = 1.15; CI: 1.13–1.17), diabetes (OR = 1.32; CI: 1.31–1.33), CVD (OR = 1.27; CI: 1.24–1.28), CLiD (OR = 1.27; CI: 1.22–1.32), HBP (OR = 1.41; CI: 1. 4–1.42), for PCI (OR = 1.06; CI: 1.04–1.08), for CND (OR = 1.12; CI: 1.09–1.13), for obesity (OR = 1.3; CI: 1.29–1.31), for CLuD (OR = 1.47; CI: 1.44–1.49) and for CKD (OR = 1.37; CI: 1.34–1.39). All these results are significant at *p* < 0.001.

## 4. Discussion

Our aim was to assess the trend in COVID-19 hospitalized and non-hospitalized cases by comorbidity and, indirectly, to further support how PA may have protected or enhanced the recovery from COVID-19 infection. In line with this aim, the main finding was that there was a statistically significant increase in the hospitalized cases with obesity and chronic lung disease throughout the pandemic period and in the interim periods studied. There was a significant increase in the non-hospitalized cases with comorbidities in the third pandemic period between December 2020 and August 2021.

In fact, it has been reported that cardiometabolic disease, such as elevated BMI or body fat, significantly increased the risk of severe COVID-19 symptoms, and that overweight and obesity, as measured by BMI, also increased this risk [18]. Some comorbidities, such as kidney disease, diabetes, hypertension and heart disease, significantly increased the likelihood of hospitalization for COVID-19 [19], and obesity and hypertension were also found to be present in more than 50% of the cases of hospitalization [20]. Most of the cases diagnosed with COVID-19 had more comorbidities than the controls, although they had fewer comorbidities when admitted to intensive care than those who were not [21]. This international evidence supports and confirms the health status of the hospitalized cases in Chile that we present here.

Regarding the evolution of the COVID-19 hospitalization cases, studies reported an increase in the per capita hospitalization rate from March to December 2020, with significant differences between the intermediate phases in subjects aged 18 to over 80 years [22], a peak in hospitalization cases in April 2020 and November 2020 [23], a decrease from May 2020 to January 2021 and then an increase from February to May 2021 in subjects aged 18 to 49 years [24], a significant increase from July to October 2020 and a significant decrease from December 2020 to January 2021 in subjects aged 26 to 66 years [25]. The increase or decrease in hospitalizations varied according to the region or country studied and was due to health control measures such as quarantines, the vaccination process and population behavior related to social order, education level, economic income, etc. Our results showed an increase in hospitalizations throughout the pandemic period studied, between June 2020 and August 2021, which is consistent with the literature reporting increases in 2020, but there are discrepancies with the comparative evidence on what happened in the months of 2021, where in our research we only found increases, whether significant or not.

However, the number of non-hospitalized cases with comorbidities increased significantly in the last pandemic period (December 2020–August 2021) of our study. This last phase could be explained by the COVID-19 vaccination process in Chile as these people, despite having comorbidities, probably did not become infected or the viral load did not exceed their immunity threshold and, among other effects of the virus, did not reach hospitalization. Although the process of mass vaccination against COVID-19 in Chile began in early February 2021 [26,27], we could hypothesize that, in the seven months of the last pandemic period of our analysis (February–August 2021), vaccination had an effect on the significant increase in the number of people who were not hospitalized but who also had comorbidities. According to our findings, people with obesity and chronic lung disease were those with a significant increase in hospital admissions throughout the pandemic research period. On the one hand, obesity causes a wide range of metabolic disorders in the body that have implications for the development of atherosclerosis and some types of cancer [28]; the accumulation of fat that characterizes obesity can also lead to the release of anti- and pro-inflammatory agents from adipocytes, including the secretion of inflammatory substances that are associated with various comorbidities such as metabolic syndrome and insulin resistance [29,30]. Other metabolic disorders caused by obesity include cardiovascular disease [31]. COVID-19 also causes cascading inflammatory processes in the lungs [32] and cardiovascular disease [33,34], and respiratory symptoms can occur in the acute phase of infection [33] and months after infection with the virus [10]. Cardiovascular problems caused by COVID-19 have even been associated with hospitalization and death [33].

In addition to the vaccination process, we believe that, in order to prevent infection by the virus and to complement the treatment and/or rehabilitation during and in a “post-pandemic” phase, this process should have an active character, involving people’s healthy lifestyles, particularly through the practice of PA. We believe in the efficacy of this non-pharmacological “treatment” because PA reduces systemic [35,36] and pulmonary [32] inflammation, mitochondrial dysfunction, reactive oxygen species (free radicals, etc. [32]), viral infections [35,36], reduces the risk of hospitalization, ICU admission and death from COVID-19 [36] and increases immunity and mitochondrial ATP resynthesis [32]. During the pandemic phase, evidence was published on how the development of cardiorespiratory fitness, an important fitness variable, reduced the likelihood of hospitalization [19,37,38], ICU admission [37] and COVID-19 mortality [37,39].

Although our study did not include data on PA variables, we would like to highlight the potential impact that PA practice can have on improving the fight against COVID-19 since it has led to a reduction in people’s ability to exercise [40] and has affected them in the periods following hospitalization for the virus [41]. An important role has been played by the “PA time bands” recommended at the time by the Chilean ministerial authority [42], which allowed citizens to conduct PA outdoors during the confinement period between 07:00 and 08:30 a.m., as well as the PA counseling provided in health centers in Chile [43], which has been shown to be a good educational tool in adults [44]. PA education should also be considered useful and necessary in the early stages of people’s lives [45] to provide guidance for the return to “normality” after a pandemic.

As we have recently discussed, this pandemic has mainly affected PA, in addition to reducing the opportunities for outdoor exercise and sports in gyms [46], so there is one aspect that has been even more affected, which is obviously the general physical health status of people who have been inactive for longer periods of time and have conducted less exercise than before the pandemic [47]. An increase in sedentary behavior in the population has already been reported in several countries, including Chile [18], while isolation at home is likely to have led to a profound decrease in PA levels and an increase in sedentary behavior [48,49]. Unfortunately, this trend has continued even after the pandemic and the implementation of containment measures. As a result, many practices that were introduced during this challenging period have been maintained, such as home delivery instead of visiting restaurants or supermarkets, teleworking and online medical consultations. On the one hand, these new practices offer more flexibility to perform daily tasks from the comfort of home. On the other hand, they may lead to reduced physical activity, such as cycling or walking to reach a destination.

A limitation of this study was the lack of access to the demographic data (sex, age, area of residence, etc.) and anthropometric characteristics (weight, height, BMI, etc.) of the hospitalized and non-hospitalized individuals. These records would have enriched the analysis in terms of calculating prevalences. One of the strengths of the present study is the large number of national cases that we were able to analyze, which, although not representative or generalizable to the Chilean population, provide valuable information on the trend curves during the pandemic period studied in Chile and offer an example from this country that can be evaluated and discussed in an international context.

## 5. Conclusions

It is possible to conclude that there was a significant trend towards an increase in hospitalized COVID-19 patients with comorbidities throughout the pandemic period studied, with obesity and chronic lung disease being the most important of these comorbidities. In addition, the association between the hospitalized and non-hospitalized cases of people with comorbidities was shown to be homogeneous, and the group of people with comorbidities in period 1 (June–August 2020) were more likely to be hospitalized compared to the subsequent pandemic periods. There was also a significant increase in non-hospitalized cases with comorbidities during the last pandemic period studied, which could be explained by the vaccination process in Chile. The vaccination against COVID-19 could be favored or reinforced by the practice of PA to reduce adverse events such as infection and hospitalization.

## Figures and Tables

**Table 1:** Cases of hospitalized COVID-19 patients by comorbidity. AMPC: average monthly percentage change; MPC: monthly percentage change; CI: confidence interval; HBP: high blood pressure; CVD: cardiovascular disease; CLuD: chronic lung disease; CHD: chronic heart disease; CKD: chronic kidney disease; CND: chronic neurological disease; ICP: immunocompromised; CLiD: chronic liver disease.

	AMPC (95% CI)	MPC (95% CI)		
Hospitalized		Period 1 ^1^	Period 2	Period 3
HBP	20.8 * (15.8–25.9)	71.5 ** (32–122.9)	15.4 *** (12.2–18.8) ^2^	9.5 (−3.9–24.8) ^6^
Diabetes	20.6 * (15.7–25.7)	69.7 ** (30.9–111.9)	15.3 *** (12.1–18.6) ^2^	9.7 (−3.6; 24.9) ^6^
Obesity	20.8 * (15.6–26.2)	73.3 ** (30.7–129.8)	11.2 ** (4.4–18.5) ^3^	16.2 *** (10.8–21.9) ^7^
Asthma	19.7 * (13.6–26.2)	62 * (16.4–125.5)	14.6 *** (10.5–18.8) ^2^	11.5 (−5.5–31.5) ^6^
CVD	17.3 * (11.7–23.1)	62.5 ** (30–103.2)	14 (−8.8–42.5) ^4^	10.1 *** (7.9–12.4) ^9^
CLuD	19.4 * (14.7–24.2)	56.9 ** (21.2–103.1)	16.5 *** (12.5–20.5) ^5^	9.4 * (0.8–18.7) ^8^
CHD	17 * (11.5–22.8)	59.1 ** (27.7–98.2)	14.3 (−8.3–42.3) ^4^	10.1 *** (7.9–12.4) ^9^
CKD	18.3 * (12.7–24.2)	72 ** (37.7–114.9)	16.1 (−7.1–45) ^4^	9.5 *** (7.3–11.8) ^9^
CND	16.3 * (11.6–21.3)	60.6 ** (23.5–108.7)	10.7 *** (7.5–13.9) ^2^	9 (−4.4–24.3) ^6^
ICP	24.5 * (19.3–29.8)	148.8 *** (90.9–224.4)	11.5 *** (8.3–14.8) ^2^	9.1 (−4.5–24.6) ^6^
CLiD	18.1 * (12.3–24.2)	65.1 ** (31.2–107.9)	13.5 (-9.8–43) ^4^	11.1 *** (8.8–13.4) ^9^

**Note:** *p*-values: * (*p* < 0.05); ** (*p* < 0.01); *** (*p* < 0.001); **Period 1:** ^1^ June 2020–August 2020; **Period 2:** ^2^ August 2020–May 2021 **//** ^3^ August 2020–Febuary 2021 **//** ^4^ August 2020–November 2020 **//** ^5^ August 2020–April 2021; **Period 3:** ^6^ May 2021–August 2021 **//** ^7^ Febuary 2021–August 2021 **//** ^8^ April 2021–August 2021 **//** ^9^ November 2020–August 2021.

**Table 2:** Non-hospitalized cases of patients with comorbidities. AMPC: average monthly percentage change; MPC: monthly percentage change; CI: confidence interval; HBP: high blood pressure; CVD: cardiovascular disease; CLuD: chronic lung disease; CHD: chronic heart disease; CKD: chronic kidney disease; CND: chronic neurological disease; ICP: immunocompromised; CLiD: chronic liver disease.

	AMPC (95% CI)	MPC (95% CI)		
Hospitalized		Period 1 ^1^	Period 2 ^2^	Period 3 ^3^
HBP	19.7 * (12.8–23.3)	55.4 ** (20.7–100.2)	8.2 (−4.6–22.8)	14.9 *** (11.8–18.1)
Diabetes	19 * (13.8–24.4)	63.8 ** (27–111.3)	8 (−4.9–22.6)	15.3 *** (12.1–18.5)
Obesity	18.1 * (13.1–23.4)	64 ** (27.7–110.6)	5.8 (−6.6–19.9)	15 *** (11.9–18.2)
Asthma	20.7 * (15–26.7)	64.5 ** (24.7–117)	9.8 (−4.4–26.2)	17.2 *** (13.7–20.7)
CVD	15.6 * (15–26.7)	52.3 ** (21.7–90.7)	6.2 (−5.1–18.8)	12.5 *** (9.8–15.3)
CLuD	17.3 * (12.7–22.1)	63.3 ** (30–105.3)	6.4 (−5.1–19.2)	13.3 *** (10.6–16.2)
CHD	16.9 * (12.4–21.7)	57 ** (25.2–97.1)	6.7 (−4.7–19.5)	13.7 *** (10.9–16.5)
CKD	16.1 * (11.8–20.5)	62.9 ** (31.7–101.5)	5.4 (−5.3–17.2)	11.9 *** (9.4–14.6)
CND	16.9 * (12.5–21.4)	65.2 ** (32.8–105.6)	4.8 (−6–16.9)	13.1 *** (10.5–15.9)
ICP	30.3 * (24.9–36)	256.4 *** (179.5–354.4)	3.4 (−8.4–16.7)	13.8 *** (10.8–16.9)
CLiD	16.5 * (11.5–21.7)	56 ** (21.6–100.2)	4.9 (−7.4–18.8)	14.1 *** (11–17.2)

**Note:** *p*-values: * (*p* < 0.05); ** (*p* < 0.01); *** (*p* < 0.001); **Period 1:** ^1^ June 2020–August 2020; **Period 2:** ^2^ August 2020–December 2020; **Period 3:** ^3^ December 2020–August-2021.

**Table 3:** Means, standard deviations (SDs), interquartile range (IQR) and *t*-values (with respective significance at the *p*-level) to compare between hospitalized and non-hospitalized cases. HBP: high blood pressure; CVD: cardiovascular disease; CLuD: chronic lung disease; CHD: chronic heart disease; CKD: chronic kidney disease; CND: chronic neurological disease; ICP: immunocompromised; CLiD: chronic liver disease.

Variables	Hospitalized		Non-Hospitalized		t (*p*)
	Mean (SD)	IQR	Mean (SD)	IQR	
HBP	237,632.1 (140,532.4)	230,780.0	74,5742.3 (414,399.1)	694,754	−7.162 ***
Diabetes	146,988 (86,796.6)	142,115.5	373,147.9 (217,732.2)	346,605	−6.6318 ***
Obesity	45,941.4 (27,334.8)	41,364.5	180,184.6 (98,749.6)	157822	−7.2693 ***
Asthma	22,956.4 (13,543.6)	21,542.5	210,070.1 (131,771.1)	217,493.5	−6.1267 ***
CVD	23,764.2 (11,529.9)	17,778.0	39,246.8 (18,772.1)	30,737.5	−8.022 ***
CLuD	29,226.2 (16,961.9)	28,756.0	38,726.2 (19,734.5)	31,846	−10.72 ***
CHD	23,856.7 (11,578.3)	17,695.5	34,092.6 (17,542.9)	28,631	−6.3285 ***
CKD	26,333.6 (12801.8)	19,466.0	27,461.6 (12,760.3)	20,488	−2.3457 *
CND	14,540.4 (6860.5)	10,585.5	20,200.4 (9970.1)	15,616.5	−6.7446 ***
ICP	14,351.1 (7612.4)	11,842.5	26,126 (14,456.3)	22,214	−6.6058 ***
CLiD	6589.8 (3369.4)	5249.5	4837.6 (2463.5)	4019	6.886 ***

**Note:** *p*-values: * (*p* < 0.05); *** (*p* < 0.001).

## Data Availability

Data can be found at https://github.com/MinCiencia/Datos-COVID19 (accessed on 7 March 2024).

## Abbreviations

The following abbreviations are used in this manuscript:

NCDs	Non-communicable Diseases
AMPC	Average Monthly Percentage Change
MPC	Monthly Percentage Change
PA	Physical Activity
BMI	Body Mass Index
CI	Confidence Interval
HBP	High Blood Pressure
CVD	Cardiovascular Disease
CLuD	Chronic Lung Disease
CHD	Chronic Heart Disease
CKD	Chronic Kidney Disease
CND	Chronic Neurological Disease
ICP	Immunocompromised
CLiD	Chronic Liver Disease

## References

[1] Liu Y.C., Kuo R.L., Shih S.R. (2020). COVID-19: The first documented coronavirus pandemic in history. Biomed. J..

[2] Lüthy I.A., Ritacco V., Kantor I.N. (2018). One hundred years after the “Spanish” flu. Medicina.

[3] Jackson J., Weiss A., Schwarzenberg A., Nelson M., Sutter K., Sutherland M. (2021). Global Economic Effects of COVID-19. Congressional Research Service. Updated 4 October 2021. https://sgp.fas.org/crs/row/R46270.pdf.

[4] Balanzá-Martínez V., Kapczinski F., de Azevedo Cardoso T., Atienza-Carbonell B., Rosa A.R., Mota J.C., De Boni R.B. (2021). The assessment of lifestyle changes during the COVID-19 pandemic using a multidimensional scale. Rev. Psiquiatr. Salud Ment..

[5] Sanyaolu A., Okorie C., Marinkovic A., Patidar R., Younis K., Desai P., Hosein Z., Padda I., Mangat J., Altaf M. (2020). Comorbidity and its impact on patients with COVID-19. SN Compr. Clin. Med..

[6] Lai F.T., Guthrie B., Wong S.Y., Yip B.H., Chung G.K., Yeoh E.K., Chung R.Y. (2019). Sex-specific intergenerational trends in morbidity burden and multimorbidity status in Hong Kong community: An age-period-cohort analysis of repeated population surveys. BMJ Open.

[7] Ryu S., Frith E., Pedisic Z., Kang M., Loprinzi P.D. (2019). Secular trends in the association between obesity and hypertension among adults in the United States, 1999–2014. Eur. J. Intern. Med..

[8] Lai S., Gao J., Zhou Z., Yang X., Xu Y., Zhou Z., Chen G. (2018). Prevalences and trends of chronic diseases in Shaanxi Province, China: Evidence from representative cross-sectional surveys in 2003, 2008 and 2013. PLoS ONE.

[9] FEN, Facultad de Economía y Negocios (Universidad de Chile) (2020). IMD Ranking: Chile Remains the Most Competitive Country in the Region. https://fen.uchile.cl/en/noticia/ver/imd-ranking-chile-remains-the-most-competitive-country-in-the-region.

[10] Fraser E. (2020). Long term respiratory complications of COVID-19. BMJ.

[11] OECD, Organisation for Economic Cooperation and Development (2021). Health at a Glance 2021: OECD Indicators. Official Report. https://www.oecd-ilibrary.org/sites/908b2da3-en/index.html?itemId=/content/component/908b2da3-en.

[12] Petermann-Rocha F., Martínez-Sanguinetti M.A., Leiva-Ordoñez A.M., Celis-Morales C. (2021). Carga global de morbilidad y mortalidad atribuible a factores de riesgo entre los años 1990 y 2019:¿ Cuál es la realidad chilena?. Rev. MÉDica Chile.

[13] Petermann F., Durán E., Labraña A.M., Martínez M.A., Leiva A.M., Garrido-Méndez A., Poblete-Valderrama F., Díaz-Martínez X., Salas C., Celis-Morales C. (2017). Factores de riesgo asociados al desarrollo de hipertensión arterial en Chile. Rev. MÉDica Chile.

[14] Leiva A.M., Martínez M.A., Petermann F., Garrido-Méndez A., Poblete-Valderrama F., Díaz-Martínez X., Celis-Morales C. (2018). Factores asociados al desarrollo de diabetes mellitus tipo 2 en Chile. Nutr. Hosp..

[15] MinSal (Ministerio de Salud de Chile; Ministry of Health of Chile), Departamento de Epidemiología-División de Planificación Sanitaria, Subsecretaría de Salud Pública (2017). Encuesta Nacional de Salud 2016–2017 Primeros Resultados. Gobernamental Report. https://www.minsal.cl/wp-content/uploads/2017/11/ENS-2016-17_PRIMEROS-RESULTADOS.pdf.

[16] MinCiencia (Ministerio de Ciencia, Tecnología, Conocimiento, e Innovación; Ministry of Science, Technology, Knowledge and Innovation) (2021). GitHub Repository from MinCiencia. https://github.com/MinCiencia.

[17] MinCiencia (Ministerio de Ciencia, Tecnología, Conocimiento, e Innovación; Ministry of Science, Technology, Knowledge and Innovation) (2021). GitHub repository of COVID-19 data from MinCiencia. Updated 4 September, 2021. COVID-19 Reports Formerly. https://www.minsal.cl/nuevo-coronavirus-2019-ncov__trashed/casos-confirmados-en-chile-covid-19/.

[18] Celis-Morales C., Salas-Bravo C., Yáñez A., Castillo M. (2020). Inactividad física y sedentarismo. La otra cara de los efectos secundarios de la Pandemia de COVID-19. Rev. MÉDica Chile.

[19] Kerrigan D.J., Brawner C.A., Ehrman J.K., Keteyian S. (2021). Cardiorespiratory fitness attenuates the impact of risk factors associated with COVID-19 hospitalization. Mayo Clin. Proc..

[20] Brawner C.A., Ehrman J.K., Bole S., Kerrigan D.J., Parikh S.S., Lewis B.K., Gindi R.M., Keteyian C., Abdul-Nour K., Keteyian S.J. (2021). Inverse relationship of maximal exercise capacity to hospitalization secondary to coronavirus disease 2019. Mayo Clin. Proc..

[21] Bergman J., Ballin M., Nordström A., Nordström P. (2021). Risk factors for COVID-19 diagnosis, hospitalization, and subsequent all-cause mortality in Sweden: A nationwide study. Eur. J. Epidemiol..

[22] Dixon B.E., Grannis S.J., Lembcke L.R., Valvi N., Roberts A.R., Embi P.J. (2021). The synchronicity of COVID-19 disparities: Statewide epidemiologic trends in SARS-CoV-2 morbidity, hospitalization, and mortality among racial minorities and in rural America. PLoS ONE.

[23] Denis F., Fontanet A., Le Douarin Y.M., Le Goff F., Jeanneau S., Lescure F.X. (2021). A self-assessment web-based app to assess trends of the COVID-19 pandemic in France: Observational study. J. Med. Internet Res..

[24] Guimarães R., Villela D., Xavier D., Saldanha R., Barcellos C., de Freitas C., Portela M. (2021). Increasing impact of COVID-19 on young adults: Evidence from hospitalisations in Brazil. Public Health.

[25] Simetin I., Svajda M., Ivanko P., Dimnjakovic J., Belavic A., Istvanovic A., Poljicanin T. (2021). COVID-19 incidence, hospitalizations and mortality trends in Croatia and school closures. Public Health.

[26] MinSal (Ministerio de Salud de Chile; Ministry of Health of Chile) (2021). Se inicia Proceso de Vacunación MASIVA Contra COVID-19. Official News. https://www.minsal.cl/se-inicia-proceso-de-vacunacion-masiva-contra-covid-19/.

[27] Gutiérrez-Jara J.P., Saracini C. (2022). Risk perception influence on vaccination program on covid-19 in chile: A mathematical model. Int. J. Environ. Res. Public Health.

[28] Irecta Najera C.A., Álvarez Gordillo G.d.C. (2016). Mecanismos moleculares de la obesidad y el rol de las adipocinas en las enfermedades metabólicas. Rev. Cuba. Investig. BiomÉDicas.

[29] Bergens O., Nilsson A., Kadi F. (2019). Cardiorespiratory fitness does not offset adiposity-related systemic inflammation in physically active older women. J. Clin. Endocrinol. Metab..

[30] Rebollo-Ramos M., Velázquez-Díaz D., Corral-Pérez J., Barany-Ruiz A., Pérez-Bey A., Fernández-Ponce C., García-Cózar F.J., Ponce-González J.G., Cuenca-García M. (2020). Aerobic fitness, Mediterranean diet and cardiometabolic risk factors in adults. Endocrinol. Diabetes Nutr. (Engl. Ed.).

[31] Boidin M., Handfield N., Ribeiro P.A., Desjardins-Crépeau L., Gagnon C., Lapierre G., Gremeaux V., Lalongé J., Nigam A., Juneau M. (2020). Obese but fit: The benefits of fitness on cognition in obese older adults. Can. J. Cardiol..

[32] Burtscher J., Millet G.P., Burtscher M. (2021). Low cardiorespiratory and mitochondrial fitness as risk factors in viral infections: Implications for COVID-19. Br. J. Sport. Med..

[33] Mihalick V.L., Canada J.M., Arena R., Abbate A., Kirkman D.L. (2021). Cardiopulmonary exercise testing during the COVID-19 pandemic. Prog. Cardiovasc. Dis..

[34] Zheng Y.Y., Ma Y.T., Zhang J.Y., Xie X. (2020). COVID-19 and the cardiovascular system. Nat. Rev. Cardiol..

[35] Salgado-Aranda R., Pérez-Castellano N., Núñez-Gil I., Orozco A.J., Torres-Esquivel N., Flores-Soler J., Chamaisse-Akari A., Mclnerney A., Vergara-Uzcategui C., Wang L. (2021). Influence of baseline physical activity as a modifying factor on COVID-19 mortality: A single-center, retrospective study. Infect. Dis. Ther..

[36] Sallis R., Young D.R., Tartof S.Y., Sallis J.F., Sall J., Li Q., Smith G.N., Cohen D.A. (2021). Physical inactivity is associated with a higher risk for severe COVID-19 outcomes: A study in 48 440 adult patients. Br. J. Sport. Med..

[37] Af Geijerstam A., Mehlig K., Börjesson M., Robertson J., Nyberg J., Adiels M., Rosengren A., Åberg M., Lissner L. (2021). Fitness, strength and severity of COVID-19: A prospective register study of 1,559,187 Swedish conscripts. BMJ Open.

[38] Brandenburg J.P., Lesser I.A., Thomson C.J., Giles L.V. (2021). Does higher self-reported cardiorespiratory fitness reduce the odds of hospitalization from COVID-19?. J. Phys. Act. Health.

[39] Christensen R.A., Arneja J., St. Cyr K., Sturrock S.L., Brooks J.D. (2021). The association of estimated cardiorespiratory fitness with COVID-19 incidence and mortality: A cohort study. PLoS ONE.

[40] Clavario P., De Marzo V., Lotti R., Barbara C., Porcile A., Russo C., Beccaria F., Bonavia M., Bottaro L.C., Caltabellotta M. (2020). Assessment of functional capacity with cardiopulmonary exercise testing in non-severe COVID-19 patients at three months follow-up. Int. J. Cardiol..

[41] Faghy M.A., Sylvester K.P., Cooper B.G., Hull J.H. (2020). Cardiopulmonary exercise testing in the COVID-19 endemic phase. Br. J. Anaesth..

[42] Ministerio de Desarrollo Social y Familia, Ministry of Social Development and Family of Chile (2021). Se inicia Proceso de Vacunación Masiva Contra COVID-19. Official News. https://eligevivirsano.gob.cl/noticias/autoridades-detallaron-alcances-de-banda-horaria-elige-vivir-sano-que-rige-desde-hoy/.

[43] Salinas J., Bello S., Chamorro H., Gonzalez C.G. (2016). Consejeria en alimentación, actividad fÍsica y tabaco: Instrumento fundamental en la practica profesional. Rev. Chil. Nutr..

[44] Berra K., Rippe J., Manson J.E. (2015). Making physical activity counseling a priority in clinical practice: The time for action is now. JAMA.

[45] Grossman D.C., Bibbins-Domingo K., Curry S.J., Barry M.J., Davidson K.W., Doubeni C.A., Epling J.W., Kemper A.R., Krist A.H., Kurth A.E. (2017). Behavioral counseling to promote a healthful diet and physical activity for cardiovascular disease prevention in adults without cardiovascular risk factors: US Preventive Services Task Force recommendation statement. JAMA.

[46] Hurtado A.F.V., Ramos O.A., Jácome S.J., Cabrera M.d.M.M. (2020). Actividad física y ejercicio en tiempos de COVID-19. Ces Med..

[47] Stanton R., To Q.G., Khalesi S., Williams S.L., Alley S.J., Thwaite T.L., Fenning A.S., Vandelanotte C. (2020). Depression, anxiety and stress during COVID-19: Associations with changes in physical activity, sleep, tobacco and alcohol use in Australian adults. Int. J. Environ. Res. Public Health.

[48] Tremblay M.S., Aubert S., Barnes J.D., Saunders T.J., Carson V., Latimer-Cheung A.E., Chastin S.F., Altenburg T.M., Chinapaw M.J. (2017). Sedentary behavior research network (SBRN)–terminology consensus project process and outcome. Int. J. Behav. Nutr. Phys. Act..

[49] Peçanha T., Goessler K.F., Roschel H., Gualano B. (2020). Social isolation during the COVID-19 pandemic can increase physical inactivity and the global burden of cardiovascular disease. Am. J.-Physiol.-Heart Circ. Physiol..

